# Chromosome-level genome assembly and annotation of a potential model organism *Gossypium arboreum* ZB-1

**DOI:** 10.1038/s41597-024-03481-z

**Published:** 2024-06-12

**Authors:** Rongnan Sun, Yuqing Wu, Xinyu Zhang, Minghua Lv, Dongliang Yu, Yuqiang Sun

**Affiliations:** Plant Genomics & Molecular Improvement of Colored Fiber Laboratory, College of Life Sciences and Medicine, Zhejiang Sci-Tech University, Hangzhou, 310008 China

**Keywords:** Plant breeding, Polyploidy in plants

## Abstract

Recent advancements in plant regeneration and synthetic polyploid creation have been documented in *Gossypium arboreum* ZB-1. These developments make ZB-1 a potential model within the *Gossypium* genus for investigating gene function and polyploidy. This work generated the sequence and annotation of the ZB-1 genome. The contig-level genome was constructed using the PacBio high-fidelity reads, encompassing 81 contigs with an N50 length of 112.12 Mb. The Hi-C data assisted the construction of the chromosome-level genome, which consists of 13 *pseudo*-chromosomes and 39 un-anchored contigs, with a total length of about 1.67 Gb. Repetitive sequences accounted for about 69.7% of the genome in length. Based on *ab initio* and evidence-based prediction, we have identified 48,021 protein-coding genes in the ZB-1 genome. Comparative genomics analysis revealed conserved gene content and arrangement between ZB-1 and *G. arboreum* SXY1. The single nucleotide polymorphism occurrence rate between ZB-1 and SXY1 was about 0.54 per 1,000 nucleotides. This study enriched the genomic resources for further exploration into cotton regeneration and polyploidy mechanisms.

## Background & Summary

Cotton (*Gossypium spp*.) is one of the most crucial fiber and oilseed crops worldwide. The *Gossypium* genus comprises about 50 species, which include 45 diploids categorized into eight genome groups (A-G and K) and seven tetraploids denoted as AD_1_-AD_7_^1,2^. The tetraploids originated from a single hybridization event between the A- and D-genome progenitors, followed by polyploidization^3^. Historically, four of these species have undergone independent domestication, *i.e*., the diploids *G. arboreum* (A_2_) and *G. herbaceum* (A_1_)^4^, as well as the tetraploids *G. hirsutum* (AD_1_) and *G. barbadence* (AD_2_)^5^. Notably, AD_1_ has emerged as the predominant species in contemporary cotton cultivation.

A_2_ is critical for investigating the mechanisms underlying natural polyploidy in the genus *Gossypium*. For an extended period, one of the major concerns in cotton biology is the origin of the A-genome in the natural tetraploids, with the controversy over whether it is from A_1_ or A_2_. In the last decade, the genome sequences of A_1_, A_2_, and several tetraploids have been successively generated^6^. With the insights gained from comparative genomics, researchers are now inclined to conclude that the ancestor (termed A_0_) of A_1_ and A_2_ is the A-genome donor^7–9^. However, as A_0_ no longer exists, A_1_ and A_2_ are still necessary substitutes for assessing the evolution of the natural cotton polyploids upon genome merge and doubling. Due to its broader distribution and, more significantly, the more profound understanding of its biology in comparison to A_1_, researchers often opt for A_2_ as the substitute progenitor when studying the evolution of natural tetraploids.

Besides, A_2_ has also frequently been used to generate synthetic cotton polyploids to investigate issues such as gene expression modulation in the early stage of polyploidy. For example, hybridization between A_2_ and *G. thurberi* (D_1_), *G. raimondii* (D_5_), and *G. bickii* (G_1_) gave rise to several hybrids and synthetic tetraploids^10,11^. Gene expression analysis of these synthetic hybrids or polyploids revealed that the subgenome expression pattern was rapidly established in the early generations of the progenies, which would then be reconciled during long-term evolution. Likewise, Ke *et al*. generated a novel cotton tetraploid between A_2_ (accession ZB-1) and *G. stocksii* (E_1_) by somatic hybridization^12^. As meiosis does not occur during somatic hybridization, the novel polyploid 2(A_2_E_1_) might be an ideal model for further exploring the transcriptional and epigenomic alteration upon genome doubling.

In addition, A_2_-derived new cotton polyploids provide valuable germplasm resources for improving agronomically important traits in cotton cultivars. For example, A_2_ and G_1_-derived tetraploids have glandular vegetative tissue and glandless seeds (without toxic gossypol)^13^, which provide novel germplasm to breed cultivars with the seeds could be more widely utilized in industrial production^14^. Likewise, by hybridizing A_2_ with E_1_ and following genome doubling, Nie and colleagues generated novel tetraploids 2(A_2_E_1_), of which the high generations produce high-strength fiber^15^. Moreover, Chen *et al*. generated a novel cotton hexaploid by hybridization between A_2_ (SXY1 or Shixiya 1) and AD_1_ (TM-1), which could be further utilized to develop the *G. arboreum*-introgressed lines to transfer the drought and *Verticillium* resistance to cultivars^16^.

In addition to serving as the progenitor for synthetic cotton polyploids in polyploidy research and breeding, A_2_ is also a potential model organism for gene function exploration in cotton species. Currently, the creation of transgenic cotton plants predominantly relies on AD_1_^6,17^. Nevertheless, ascribed to the doubled genomes in AD_1_, each allele is associated with four haplotypes that are similar in sequence. The complexity of the genetic background poses a challenge in gene manipulation in AD_1_, such as the risk of off-target effects in CRISPR/Cas9-mediated genome editing. Furthermore, the gene duplication resulting from polyploidy also adds to the complexity of evaluating gene functions in AD_1_. For instance, several studies have highlighted that subfunctionalization and neofunctionalization of duplicated genes frequently occur after cotton polyploidization^11,18,19^. Consequently, diploids such as A_2_ are better models for characterizing gene functions in cotton species.

However, the method of regenerating plants by somatic embryogenesis has yet to be established for most cotton species, which significantly hindered the transgenic study in cotton cells. Recently, we have achieved plant regeneration using A_2_ (ZB-1) with alternative solid-liquid culture method^20^. The callus was able to be induced from different explants (hypocotyl, root, and cotyledon), and the regenerated plants could be regularly grown to maturity in soil or be grafted onto A_2_ seedlings. Upon further optimization, ZB-1 will become a readily available tool for the functional analysis of cotton genes.

**Fig. 1:**
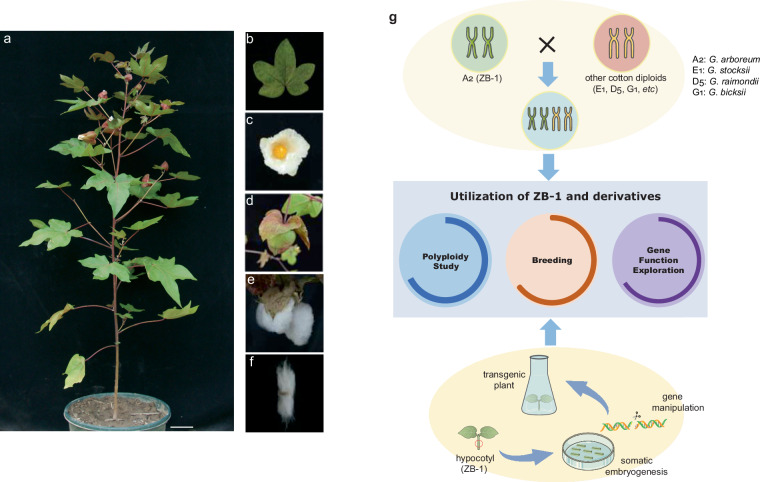
*Gossypium arboreum* ZB-1 and its utilization in cotton biology study. a-f indicate the morphological characteristics of ZB-1: (**a**) whole plant, (**b**) leaf, (**c**) flower, (**d**) boll, (**e**) opening boll, and (**f**) fiber. (**g**) indicates the potential utilization of ZB-1. *G. arboreum* accessions including ZB-1 could hybrid with many other cotton diploids and generate synthetic polyploids. In addition, ZB-1 could produce regenerated cotton plants via somatic embryogenesis, which is essential for further manipulating the genes and construct the transgenic plants. ZB-1 derived novel polyploid and transgenic plants will help the future study on cotton polyploidy, gene function, and breeding.

Taken together, the accession ZB-1 with A_2_ genome is an essential material for the studies on cotton polyploidy and gene function exploration (Fig. 1). At the same time, we have noticed that although some genome assemblies of A_2_ are available now and tens of A_2_ accessions have been resequenced, the chromosome-level A_2_ genomes were almost exclusively generated from the accession SXY1^7,21–24^. Therefore, a high-quality genome assembly of ZB-1 is necessary to provide additional genomic information for studies on polyploidy, gene manipulation, and the mechanisms underlying the regeneration of cotton plants.

In this work, we constructed a chromosome-level genome assembly of *G. arboreum* ZB-1. The contig-level genome assembly was generated using PacBio HiFi reads. The Hi-C data was used to scaffold the contigs into *pseudo*-chromosomes. Four transcriptomes of ZB-1 (boll, flower, sepal, and stem) were generated to assist the gene identification. The final gene set was generated by integrating the results from *ab initio* prediction, homologs-based gene annotation, and transcripts-derived gene identification using the PASA pipeline. The completeness of the genome assembly was evaluated by BUSCO assessment and the mapping back of the short reads, which showed the high quality of the provided genome assembly of ZB-1. Moreover, a comparative analysis was carried out between the assemblies of ZB-1 and SXY1, revealing a large number of single nucleotide polymorphisms (SNPs), suggesting this genome assembly is necessary for further studies using ZB-1 as a test material.

## Methods

### Plant material collection

*G. arboreum* ZB-1 was planted in the experimental field on the campus of Zhejiang Sci-Tech University in Hangzhou, Zhejiang Province, China, in May 2022. The tender leaves (the third to fifth), bolls (10 days post anthesis), flowers (bloom day, including petals, stamens and pistils), sepals, and stems were harvested from at least five adult plants and pooled. All the tissues were snap-frozen in liquid nitrogen and stored at −80 °C until use.

### DNA library construction and genome sequencing

The genomic DNA (gDNA) was extracted from the tender leaves using the CTAB (cetyltrimethylammonium bromide) method. After chilling in liquid nitrogen, 20 mg leaves were ground with steel balls in a 2 mL polypropylene tube. 600 μL pre-heated (65 °C) CTAB buffer (2% w/v CTAB, 100 mM Tris-HCl, 20 mM EDTA, 1.5 M NaCl, pH 8) was added into the tube and thoroughly vortexed. The tube was placed in a 65 °C water bath for 1 h. The homogenate was then centrifuged for 10 min at 12,000 rpm. The supernatant was transferred to a new tube, and 5 μL of RNase A solution was added. After incubating at 37 °C for 20 min, an equal volume of phenol/chloroform/isoamyl alcohol (25:24:1) was added and mixed. The sample was centrifuged for 10 min at 12,000 rpm. The upper aqueous phase was transferred to a new tube, and repeat this extraction once. Cold isopropanol was added with a volume of about two-thirds of the upper phase. The sample was gently mixed and incubated at −20 °C for 30 min. Next, the sample was centrifuged for 10 min at 12,000 rpm at room temperature, and the pellet was washed using 500 μL ice-cold 70% ethanol. Decant the ethanol and naturally dry the pellet to remove the ethanol residual. Finally, about 20 uL TE buffer (10 mM Tris, pH 8, 1 mM EDTA) was added to dissolve the DNA pellet.

#### Illumina sequencing

For Illumina short-read sequencing, the purified gDNA was randomly sheared into fragments by a Covaris M220 Ultrasonicator (Covaris, USA). Subsequently, a library with an average length of about 350 bp for the inserted fragments was constructed according to the recommended protocols of the Illumina TruSeq DNA Sample Prep Kits. Qubit 2.0 Fluorometer (ThermoFisher Scientific, USA) was used to quantify the dsDNA of the library, which was then diluted to 1 ng/μL. Agilent 2100 Bioanalyzer (Agilent, USA) was used to assess the integrity and size of the inserted fragments. A final concentration of the library was determined with QuantStudio 3 Real-Time PCR Instrument (Thermo Fisher Scientific, USA) (>2 nM). The library was sequenced using the Novaseq 6000 platform (Illumina, USA) and a program of pair-end 150 bp. Quality control of the raw data was performed using Fastp (v0.21.0)^25^. The read pairs were removed if the ambiguous base (N) exceeds 10% or the low-quality bases (Q < 5) exceed 20% of a single read in length. The generated raw data contained about 101.03 Gb paired short reads, with 99.91% retained after quality control (Table 1).

**Table 1:** Statistics of the data generated for the genome assembly and annotation of *Gossypium arboreum* ZB-1.

Platform	Strategy	Sample	Molecule	Clean data	Coverage*	Usage	Accession Number
Illumina NovaSeq	PE150	Leaf	DNA	101 Gb	60.6×	correction	SRR27009933
PacBio HiFi	CCS	Leaf	DNA	69 Gb	41.4×	contig-level assembly	SRR27009931
Illumina Hi-C	PE150	Leaf	DNA	3.8 Gb	2.3×	chromosome-level assembly	SRR27009932
Illumina NovaSeq	PE150	Stem	RNA	6.3 Gb	—	gene identification	SRR27009934
Illumina NovaSeq	PE150	Sepal	RNA	6.7 Gb	—	gene identification	SRR27009935
Illumina NovaSeq	PE150	Flower	RNA	6.1 Gb	—	gene identification	SRR27009936
Illumina NovaSeq	PE150	Boll	RNA	6.7 Gb	—	gene identification	SRR27009937

*the coverage was estimated based on the final version of genome assembly.

#### Pacbio sequencing

A HiFi library was constructed using the SMRTbell Template Prep Kit (Pacific Biosciences, USA). The high molecular weight DNA fragments (*ca*. 20 Kb) were isolated and purified from the g-TUBE (Covaris, USA) sheared gDNA sample using the AMPure beads (Beckman Coulter, USA), followed by damage repair, end-repair/A-tailing and hairpin adapter ligation according to the manufacturer’s instructions. The SMRTbell library was sequenced on the Pacbio Sequel II platform with the Circular Consensus Sequencing (CCS) model. The HiFi reads were generated using SMRT Link software (v12.0) with default settings. About 4.01 M HiFi reads were finally acquired, with a total length and average length of about 71 Gb and 17.7 kb, respectively (Table 1).

### Hi-C library construction and sequencing

The Hi-C library was constructed according to the method previously described by Belton *et al*. with certain modifications^26^. Tender leaves of ZB-1 were ground with liquid nitrogen and crosslinked by 4% formaldehyde at room temperature for 30 mins. 2. 5 M glycine was added to quench the crosslinking reaction. The pellet was centrifuged at 2,500 rpm at 4 °C and resuspended with the lysis buffer (1 M Tris-HCl, pH 8, 1 M NaCl, 10% CA-630, and 13 units protease inhibitor). The supernatant was centrifuged at 5,000 rpm at room temperature. The pellet was washed twice using the ice-cold NEB buffer and centrifuged again. Then, the nuclei were resuspended by the NEB buffer, solubilized with dilute sodium dodecyl sulphate (SDS), and incubated at 65 °C for 10 mins. Triton X-100 quenched SDS, and the crosslinked chromatin was digested overnight by the restriction enzyme *DPN II* (400 units, 37 °C). The 5’ overhangs were filled in with biotinylated residues (biotin-14-dCTP), and the proximate blunted ends were ligated. Biotin was removed from the un-ligated ends using T4 DNA polymerase. The ligated DNA was then sheared with the Covaris instrument, and the fragments with an average size of 350 bp were purified via agarose gel electrophoresis and gel elution. After end repairing by the mixture of T4 DNA polymerase, T4 polynucleotide kinase, and Klenow DNA polymerase, the biotin-labelled DNA fragments were pulled down using the streptavidin C1 magnetic beads. The library was then constructed with a standard protocol and sequenced on the Illumina Novaseq 6000 platform with 2 × 150 bp paired-end reads (PE150).

About 3.80 Gb short reads were generated from Hi-C library sequencing, with 3.78 Gb (99.6%) retained after removing the low-quality reads (Table 1). Further quality control was mainly carried out with HICUP^27^. The truncated sequences were identified and mapped with hicup_truncater and hicup_mapper. The re-ligated and same circularised sequences were removed by hicup_filter, and the PCR-raised duplication was filtered using hicup_deduplicator. As a result, 2,494,732 unique di-Tags were obtained, with the effect rate of Hi-C sequencing data about 23.32%.

### RNA library construction and sequencing

Total RNA was isolated from the tissue samples (boll, flower, sepal, and stem) using the RNAprep Pure Plant Plus Kit (DP441, TIANGEN) and purified with RNase-free DNase I. The sequencing libraries were constructed using NEB Next Ultra RNA Library Prep Kit for Illumina (New England Biolabs, USA). The mRNAs were enriched using the oligo(dT) attached beads, and the cDNAs were synthesized using Superscript II reverse transcriptase. Fragmented cDNAs with a length of around 350 bp were used for sequencing library construction with standard protocol. The constructed libraries were sequenced on Novaseq 6000 with the PE150 strategy.

About 6.75 Gb, 6.16 Gb, 6.82 Gb, and 6.36 Gb raw data were generated from sequencing of the libraries of the boll, flower, sepal, and stem, respectively. After quality control, about 6.1 to 6.7 Gb clean data were retained for further analysis, with their effective ratio ranging from 98.21% to 98.93% (Table 1).

### Genome assembly

**Fig. 2:**
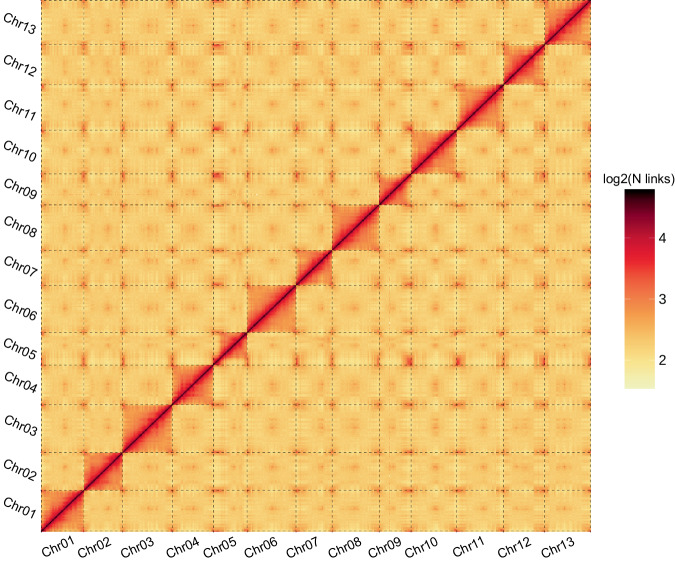
Heat map of Hi-C interactions in *Gossypium arboreum* ZB-1. The color indicates the number of Hi-C reads supporting the interactions.

The Pacbio sequencing generated HiFi reads were used to construct the contig-level genome assembly with Hifiasm (v0.13.0-R307)^28^. 81 contigs were generated with a total length of 1,667.63 Mb. The length of the contigs ranged from 3.8 kb to 149.26 Mb, with the N50 and N90 lengths of 112.12 Mb and 41.97 Mb, respectively. The Hi-C data was then used to construct the chromosome-level assembly with the ALLHiC pipeline^29^. The tool juicebox (v2.20.00) was used for a manual check and accuracy of the generated assembly (Fig. 2)^30^. 52 scaffolds were finally acquired, with the N50 and N90 lengths of 137.59 Mb and 102.94 Mb, respectively (Table 2). The total length of 13 *pseudo*-chromosomes was 1,663.66 Mb, accounting for 99.77% of the entire assembly in length (Table 3).

**Table 2:** Statistics of the *Gossypium arboreum* ZB-1 genome assembly.

Features	Metric
**Contig-level assembly**	
Number of contigs	81
Total length of contigs	1,667,622,968 bp
Longest contig	149,263,857 bp
Contig N50	112,123,399 bp (contig number = 7)
Contig N60	97,033,785 bp (contig number = 9)
Contig N70	63,206,004 bp (contig number = 11)
Contig N80	53,605,035 bp (contig number = 14)
Contig N90	41,967,933 bp (contig number = 17)
**Chromosome-level assembly**	
Number of scaffolds	52
Total length of scaffolds	1,667,433,246 bp
Longest scaffold	149,494,948 bp
Scaffold N50	137,589,794 bp (scaffold number = 6)
Scaffold N60	125,121,100 bp (scaffold number = 8)
Scaffold N70	124,213,643 bp (scaffold number = 9)
Scaffold N80	117,719,896 bp (scaffold number = 10)
Scaffold N90	102,940,630 bp (scaffold number = 12)
GC content	35.30%

**Table 3:** Statistics of the final genome assembly of *Gossypium arboreum* ZB-1.

Chromosome	Length (bp)	N base number
Chr01	128952358	0
Chr02	117719896	0
Chr03	149494948	500
Chr04	125121100	100
Chr05	102940630	700
Chr06	147578419	300
Chr07	108479559	100
Chr08	144651629	100
Chr09	95605883	200
Chr10	138868839	100
Chr11	142448263	400
Chr12	124213643	200
Chr13	137589794	100
Total anchored	1663664961 (99.77%)	2800
Unanchored	3768285 (0.23%)	0

### Genome annotation and gene function prediction

#### Annotation of repetitive sequences

A hybrid strategy was used to identify the repetitive sequences in the ZB-1 genome. The tandem repeats were predicted using TRF (v4.09) (Match = 2, Mismatch = 7, Delta = 7, PM = 80, PI = 10, MaxPeriod = 2000)^31^. The homology-based prediction was carried out using the Repbase database (v202101) and the software RepeatMasker (v4.1.0) (-a -nolow -no_is -norna) and RepeatProteinmask (v4.1.0) (-noLowSimple -pvalue 0.0001 -engine ncbi) (http://www.repeatmasker.org/)^32^. The *de novo* repetitive elements database was built with LTR_FINDER (v1.06) (-C -w 2)^33^, RepeatScout (v1.0.5)^34^, and RepeatModeler (v2.0.1) (-engine ncbi), then all repeat sequences with lengths greater than 100 bp and gap ‘N’ less than 5% constituted the raw transposable element (TE) library. This TE library was combined with the results from the homology search against Repbase to construct the custom library using Uclust (-id 80)^35^, and supplied to RepeatMasker for DNA-level repeat identification.

A total of 1,161.62 Mb of repetitive sequences were identified in the ZB-1 genome, representing 69.7% of the entire assembly in length. Apart from the tandem repeats (61.4 Mb, 3.68%), the other repetitive sequences were mainly classified into long terminal repeats (LTR), DNA, and long interspersed nuclear elements (LINE) (Table 4).

**Table 4:** Summary of the annotated repetitive sequences in *Gossypium arboreum* ZB-1.

Type	Length (bp)	% in genome
DNA	10113171	0.60651
LINE	2663587	0.15974
SINE	2214	0.00013
LTR	1131150991	67.83786
Satellite	266333	0.01597
Unknown	10153547	0.60893
Total	1148096143	68.85410

Long terminal repeat, LTR; Long interspersed nuclear element, LINE; Short interspersed nuclear element, SINE.

#### Annotation of coding genes

The coding genes were identified by integrating the results from *ab initio* prediction, homology-based prediction, and cDNA-assisted prediction. Augustus (v3.2.3) (--genemodel = complete,--noInFrameStop = true)^36^, GlimmerHMM (v3.0.4)^37^, SNAP (v2013.11.29)^38^, Geneid (v1.4), and Genscan (v1.0) were used for *ab initio* prediction^39^. The predicted proteomes of closely related species, *i.e*., A_2_ (‘SXY1’ genome WHU-updated v1)^7^, E_1_ (ZSTU_v1)^40^, D_5_ (JGI_v2_a2.1)^41^, AD_1_ (‘TM-1’ genome NBI_v1.1)^42^, and *Theobroma cacao*^43^, were downloaded from cottongen (https://www.cottongen.org/) and Ensembl Genomes (http://ensemblgenomes.org/) and used for homology-based gene prediction. These protein sequences were aligned to the ZB-1 genome assembly using TBLASTN (v2.2.26) (-F T -e 1e-5), and the matching ones were further aligned to the genome with GeneWise (v2.4.1) for an accurate prediction of the gene structures^44,45^.

In addition, RNA-seq data of the ZB-1 tissues (leaf, boll, flower, sepal, and stem) were used to assist the gene prediction. Notably, the RNA-seq data of ZB-1 seedling leaves were generated and submitted to the public database by our previous work^40^, including SRR13933598, SRR13933601, and SRR13933602. Trinity (v2.1.1) (--normalize_reads --full_cleanup –min_glue 2 –min_kmer_cov 2 --KMER_SIZE 25) was used to generate *de novo* transcripts^46^. In addition, the short reads from RNA-seq were aligned to the ZB-1 genome using Hisat2 (v2.2.1), and the resulting bam files were used for re-constructing the gene models using Stringtie (v1.3.3)^47,48^.

The non-redundant gene annotation was then generated by merging the results derived from three methods with EvidenceModeler (EVM, v1.1.1) (--segmentSize 200000 --overlapSize 20000 --min_intron_length 20) and was improved by using PASA pipeline and manual check^49^.

This study finally identified 50,058 loci of coding genes, including 48,236 genes of high confidence and 1,822 pseudogenes (Table 5). The majority of coding genes (48,021) were found within the constructed chromosomes, in contrast to a small number (215) of genes in the unanchored contigs. The high confidential genes spanned an average of 2,620 bp in the genome, with an average exon number of 4.3 and an average coding sequence (CDS) length of 1,047 bp.

**Table 5:** Prediction of the coding genes in *Gossypium arboreum* ZB-1 using different strategies.

Strategy	Gene set	Number	Average gene length (bp)	Average CDS length (bp)	Average exons per gene	Average exon length (bp)	Average intron length (bp)
*de novo*	Augustus	53962	2072	954	4.1	234	363
	GlimmerHMM	104037	12984	550	2.9	191	6629
	SNAP	34386	11431	530	3.7	143	4036
	Geneid	86341	4408	639	3.7	173	1397
	Genscan	58343	16689	942	5.1	187	3888
homology-based	*Gossypium arboreum*	48178	2440	955	4.1	235	486
	*Gossypium stocksii*	59129	2188	821	3.6	231	536
	*Theobroma cacao*	34216	2766	1148	4.7	243	433
	*Gossypium hirsutum*	48347	2366	940	4.0	236	479
	*Gossypium raimondii*	45763	2295	959	4.0	238	440
cDNA-based	transcripts	54101	4115	1654	5.5	302	549
	PASA	31005	2478	1047	4.7	223	388
EVM		60626	2371	900	4.0	228	499
PASA-update*		60557	2328	896	3.9	230	494
Final		48236	2620	1047	4.3	245	476

*Only the genes in the PASA-updated collection contain the untranslated regions.

*contains the UTR.

#### Functional annotation

Functional annotation of the coding genes was performed by homology search against the non-redundant protein database (NR) in NCBI using BLASTP (cutoff evalue 1e-4 and identity 25%). The domain architectures and Gene Ontology annotations of the predicted proteins were characterized by Interproscan (v5.35–74.0) (-appl ProDom, SMART, ProSiteProfiles, PRINTS, Pfam, Panther -iprlookup -dp -goterms) and online service of eggNOG (http://eggnog-mapper.embl.de/)(--evalue 0.001 --score 60 --pident 35)^50,51^. The combined analysis assigned tentative functions for about 99% of the predicted coding genes. 17,700 genes were mapped to the KEGG pathways, and a similar number of genes (17,774) were assigned GO terms.

#### Annotation of noncoding RNAs

The tRNAs in the ZB-1 genome were identified using tRNAscan-SE (v2.0.12)^52^, and the rRNA genes were identified via homology search with BLASTN (v2.2.26) (-e 1e-10). In addition, Rfam (v14.1) and INFERNAL (v1.1.3) (http://infernal.janelia.org/) were used to predict the miRNAs and snRNAs^53,54^. 274 miRNA genes and 1,369 tRNA genes were identified, as well as 14,383 rRNA and 7,467 snRNA loci (Table 6).

**Table 6:** Summary of the annotated noncoding RNA genes in *Gossypium arboreum* ZB-1.

ncRNA	Type	Number	Average length (bp)	Total length (bp)	% of genome
miRNA	—	274	127.1	34827	0.00209
tRNA	—	1369	75.1	102843	0.00617
rRNA	18S	519	1673.5	868561	0.05209
	28S	1875	141.2	264775	0.01588
	5.8S	469	167.2	78425	0.00470
	5S	11520	120.6	1389299	0.08332
snRNA	CD-box	7268	106.7	775454	0.04651
	HACA-box	51	132	6733	0.00040
	splicing	145	149	21606	0.00130

### Comparative genomics analyses

#### Genomic synteny

**Fig. 3:**
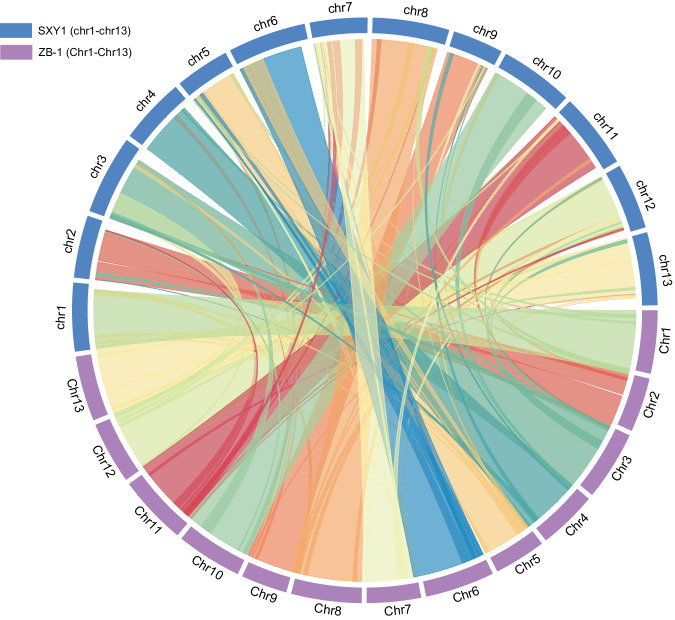
Genomic synteny between *Gossypium arboreum* accessions ZB-1 and SXY1.The collinear regions were determined based on the similarity of coding genes, with each collinear block contained at least ten genes.

The predicted proteomes of ZB-1 and SXY1 (WHU-updated v1) were compared using BLASTP (-evalue 1e-5, cutoff identity 50%, coverage 50%). Based on the homology analysis, MCScanX (-s 10) was then used to identify the collinear blocks^55^, with the result visualized using the R package RIdeogram (0.2.2)^56^. Extensive genomic synteny was observed between the genomes of these two A_2_ accessions (Fig. 3), i*.e*., 99.1% of the coding genes (including pseudogenes) were encoded by the collinear blocks.

#### SNP calling

**Fig. 4:**
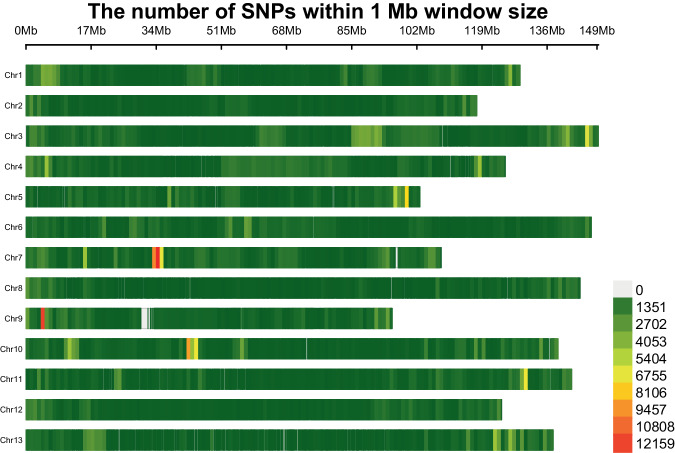
A graphic view of the single nucleotide polymorphisms between *Gossypium arboreum* SXY1 and ZB-1. Single nucleotide polymorphisms (SNPs) were identified through genome alignment between the SXY1 and ZB-1 strains, and their distribution across the chromosomes of ZB-1 is presented here. The count of SNPs was determined within a window size of 1 Mb. The color (green to red) indicates the density (low to high) of SNP in the chromosomes.

Discovery of the SNPs between ZB-1 and SXY1 was carried out using the MUMmer tools (v4.0.0)^57^. The script *nucmer* was used to align the two genome sequences, and the script *delta-filter* was subsequently used to remove the redundancy in the alignments (−1 -q -r). The structural variations were then called using the script *show-snaps* (-C), and the result was displayed using the R package of CMplot (v4.5.0)^58^. The overall rate of the SNP occurrence was about 0.53 in every 1,000 nucleotides (Fig. 4). Specifically, 19,017 SNPs were identified in the coding regions (cSNPs), with the occurrence rate of about 0.38 in 1,000 nucleotides.

## Data Records

All the high-throughput sequencing data generated in this work were available through NCBI with the project PRJNA935667. The clean data generated by sequencing the genome libraries were deposited in the Sequence Read Archive (SRA) under the accession numbers SRR27009933 (Illumina PE150) and SRR27009931 (Pacbio CCS)^59,60^. The data generated from sequencing of the Hi-C library was deposited under SRR27009932^61^. The RNA-seq data of the boll, flower, sepal, and stem could be retrieved from the SRA with the accession numbers SRR27009934-SRR27009937^62–65^.

The assembled genome sequence of *G. arboreum* ZB-1, together with the information on the coding gene structures, has been deposited in GenBank under the accession number JARKNE000000000^66^. The annotation of noncoding genes and repeat sequence, as well as the gene function prediction, were available in the Figshare database (10.6084/m9.figshare.24736338)^67^.

## Technical Validation

### Evaluation of the completeness and quality of the genome assembly

#### Mapping the short reads to the genome

The short reads generated from Illumina sequencing of the genome library were aligned to the assembly using BWA (v0.7.17)^68^, which showed that 99.22% of the reads were mapped to 99.95% of the genome sequence. The average depth of short reads mapping was about 50, with 99.73% of the genomic regions covered by at least four reads, suggesting high concordance and completeness of the ZB-1 genome assembly (Table 7).

**Table 7:** Statistics of the short reads mapping to the final genome assembly.

	Feature	Value
Reads	Mapping rate	99.22%
Genome	Average sequencing depth	49.96×
	Coverage	99.95%
	Coverage at least 4×	99.73%
	Coverage at least 10×	98.95%
	Coverage at least 20×	96.80%

Based on the result of short reads alignment, SNP calling was performed using samtools (v1.7)^69^, which showed an extremely low rate of heterozygosis SNP (0.000563%) and rare homology SNP (<10). In addition, the *k*-mer based method Merqury was also used to estimate the quality of the genome assembly^70^, which showed high base accuracy of the genome assembly (Q-value about 50).

#### Mapping of RNA-seq data

The RNA-seq data generated by this work, *i.e*., for the ZB-1 samples of flower, sepal, stem, and boll, were aligned to the genome assembly using HiSat2 (v2.2.1) with default settings^71^. According to the statistics of the alignment, 94.5% (boll), 96.2% (flower), 91.9% (sepal), and 94.4% (stem) of the properly paired short reads from RNA-seq could be mapped to the *pseudo*-chromosomes.

#### BUSCO assessment

The BUSCO (Benchmarking Universal Single-Copy Orthologs) method was also used to evaluate the completeness of the genome assembly^72,73^. The ZB-1 genome assembly captured 99.4% of the BUSCO 1614 reference gene set (embryophyta_odb10), indicating the completeness of the ZB-1 genome assembly (Table 8).

**Table 8:** Statistics of the BUSCO assessment.

	Number of genes in BUSCOs	Percentage of BUSCOs
Complete	1604	99.38%
Complete and single-copy	1497	92.75%
Complete and duplicated	107	6.63%
Fragmented	5	0.31%
Missing	5	0.31%

#### Evaluation of the genome annotation

**Fig. 5:**
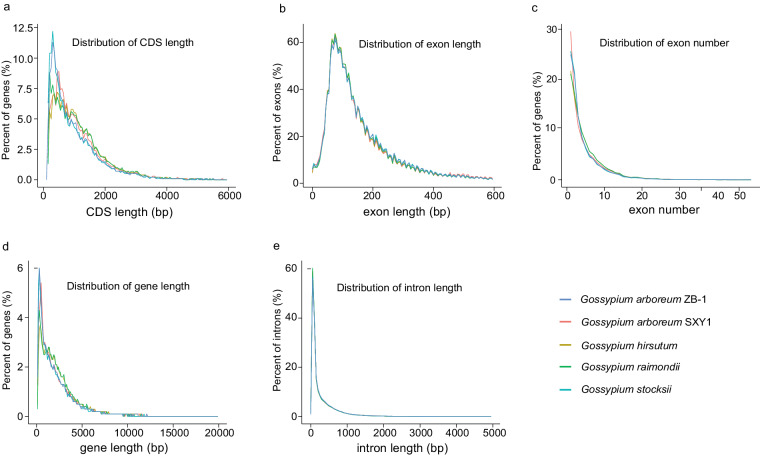
Comparison of the gene features among *Gossypium* species. a-e indicate the distribution of the (**a**) CDS length, (**b**) exon length, (**c**) exon number, (**d**) gene length, and (**e**) intron length. The untranslated regions were not included when calculating the exon length and number. The gene length was estimated by the length of the genomic region from the translation initiation site to the translation termination site.

The genomic features of the coding genes were compared between *G. arboreum* ZB-1 and closely related species within the genus *Gossypium*, including *G. arboreum* SXY1, *G. hirsutum*, *G. raimondii*, and *G. stocksii*, with the versions of their genome assemblies as aforementioned. According to the distribution of the exon number and the length of CDS, exon, gene, and intron, the gene features of ZB-1 were highly consistent with other cotton species (Fig. 5), suggesting the overall high accuracy of the gene annotation. The predicted proteome of ZB-1 was also subjected to a search against the BUSCO 1614 reference gene set (embryophyta_odb10) using HMMER (v3.4) (http://hmmer.org/). With the recommended cutoff by BUSCO, this assessment showed that 98.4% of the reference genes have been annotated in this study.

### Evaluation of the SNP calling

About 95.1 Gb of short reads from sequencing the genome library of SXY1 were downloaded from SRA with the accession number SRR13061943^74^. About 97.5% of the reads were retained after quality control using Trimmomatic (v0.33) (SLIDINGWINDOW:5:20 LEADING:5 TRAILING:5 MINLEN:50)^75^. 97.1% of the clean data were aligned to the ZB-1 genome sequence using BWA (v0.7.17) with default settings. GATK (v4.0.5.1) tools *MarkDuplicates* and *HaplotypeCaller* were used to remove the redundancy raised by PCR amplification and identify the structure variations (default parameters), respectively^76^. According to the result, 88.1% (16,752/19,017) of the cSNPs identified from genome sequence alignment (by MUMMER) were validated by the short reads, suggesting high accuracy of the estimation of the SNP occurrence rate between SXY1 and ZB-1 and also high quality of the ZB-1 genome assembly.

## Data Availability

All the bioinformatics tools were used according to the users’ manuals. Otherwise specified in the context, default settings were used during data processing. No specific custom code has been developed in this study.
